# E-Cigarette Surveillance With Social Media Data: Social Bots, Emerging Topics, and Trends

**DOI:** 10.2196/publichealth.8641

**Published:** 2017-12-20

**Authors:** Jon-Patrick Allem, Emilio Ferrara, Sree Priyanka Uppu, Tess Boley Cruz, Jennifer B Unger

**Affiliations:** ^1^ Keck School of Medicine University of Southern California Los Angeles, CA United States; ^2^ Information Sciences Institute University of Southern California Los Angeles, CA United States; ^3^ Department of Computer Science University of Southern California Los Angeles, CA United States

**Keywords:** electronic cigarettes, vaping, Twitter, social media, social bots, electronic nicotine delivery system, infoveillance

## Abstract

**Background:**

As e-cigarette use rapidly increases in popularity, data from online social systems (Twitter, Instagram, Google Web Search) can be used to capture and describe the social and environmental context in which individuals use, perceive, and are marketed this tobacco product. Social media data may serve as a massive focus group where people organically discuss e-cigarettes unprimed by a researcher, without instrument bias, captured in near real time and at low costs.

**Objective:**

This study documents e-cigarette–related discussions on Twitter, describing themes of conversations and locations where Twitter users often discuss e-cigarettes, to identify priority areas for e-cigarette education campaigns. Additionally, this study demonstrates the importance of distinguishing between social bots and human users when attempting to understand public health–related behaviors and attitudes.

**Methods:**

E-cigarette–related posts on Twitter (N=6,185,153) were collected from December 24, 2016, to April 21, 2017. Techniques drawn from network science were used to determine discussions of e-cigarettes by describing which hashtags co-occur (concept clusters) in a Twitter network. Posts and metadata were used to describe where geographically e-cigarette–related discussions in the United States occurred. Machine learning models were used to distinguish between Twitter posts reflecting attitudes and behaviors of genuine human users from those of social bots. Odds ratios were computed from 2x2 contingency tables to detect if hashtags varied by source (social bot vs human user) using the Fisher exact test to determine statistical significance.

**Results:**

Clusters found in the corpus of hashtags from human users included behaviors (eg, #vaping), vaping identity (eg, #vapelife), and vaping community (eg, #vapenation). Additional clusters included products (eg, #eliquids), dual tobacco use (eg, #hookah), and polysubstance use (eg, #marijuana). Clusters found in the corpus of hashtags from social bots included health (eg, #health), smoking cessation (eg, #quitsmoking), and new products (eg, #ismog). Social bots were significantly more likely to post hashtags that referenced smoking cessation and new products compared to human users. The volume of tweets was highest in the Mid-Atlantic (eg, Pennsylvania, New Jersey, Maryland, and New York), followed by the West Coast and Southwest (eg, California, Arizona and Nevada).

**Conclusions:**

Social media data may be used to complement and extend the surveillance of health behaviors including tobacco product use. Public health researchers could harness these data and methods to identify new products or devices. Furthermore, findings from this study demonstrate the importance of distinguishing between Twitter posts from social bots and humans when attempting to understand attitudes and behaviors. Social bots may be used to perpetuate the idea that e-cigarettes are helpful in cessation and to promote new products as they enter the marketplace.

## Introduction

Electronic cigarettes (e-cigarettes) have climbed in popularity in the United States and elsewhere [1-6]. As e-cigarette use (vaping) rapidly becomes more prevalent, data from online social systems (eg, Google Web Search, Instagram, Twitter, YouTube) can be used to capture and describe the social and environmental context in which individuals use, perceive, and are marketed this tobacco product [7]. These data may serve as a massive focus group allowing for people to organically discuss e-cigarettes unprimed by a researcher, without instrument bias, captured in near real time and at low costs [8].

Internet searches (Google Web Search) for e-cigarette–related terms increased by 450% from 2010 to 2014 in the United States with search volume for e-cigarettes greater in coastal areas (California and New York) in 2010 before becoming more uniformly searched across the contiguous United States in 2014 [9]. Searches for terms indicative of purchasing e-cigarettes have outpaced searches indicative of interest in health concerns or smoking cessation [9]. In a study analyzing e-cigarette–related posts on Instagram, Chu and colleagues [10] reported that images often showed “cloud chasing” (ie, large clouds of aerosol being blown) and “hand checks” (ie, e-cigarette device paired with specific e-juice bottle all held in one hand), suggesting these are appealing characteristics of this emerging tobacco product.

Twitter has been used in tobacco control research with studies showing how tobacco education campaigns can be informed by monitoring tweets [11,12] and which e-cigarette–related messages are likely to spread on Twitter [13], among other studies [14-21]. Ayers and colleagues [22] recently analyzed a sample of e-cigarette–related tweets and reported that social image was the most identified reason for e-cigarette use in 2015. Other identified reasons for e-cigarette use included quitting combustible cigarettes and use indoors [22].

In this study, we demonstrate the feasibility of a Twitter-based infoveillance [7] methodology to document and describe e-cigarette–related conversations on Twitter. We used social network analyses to identify discussions of e-cigarettes by describing which hashtags co-occur in a massive Twitter network. Twitter users use hashtags (ie, terms prepended by the hash mark #) to indicate the context, emotions, or subject matter related to a post. Hashtags serve as a marker for the content of posts that allows users to search for and see posts of other Twitter users even if they do not follow them. Multiple hashtags can be adopted in a single post. When 2 hashtags co-occur in the same post, one can infer that they are related. Building the network of co-occurrence of hashtags (ie, hashtag network) will illustrate concept clusters giving us insights to e-cigarette–related discussions by individuals in their own words. This clustering allows us to see underlying dimensions of meaning that might not otherwise be possible in complex data.

We also used posts and metadata from Twitter to describe where geographically e-cigarette–related discussions in the United States occur to identify priority areas for e-cigarette education campaigns. Additionally, this study builds on earlier work [23,24] and demonstrates the importance of removing social bots (ie, computer algorithms designed to automatically produce content and engage with legitimate human accounts on Twitter) from Twitter data when attempting to understand public health–related behaviors and attitudes. Taken together, findings from this study should inform tobacco control and demonstrate the utility in using Twitter data in enhancing surveillance of health behaviors in general and e-cigarette use.

## Methods

Data were obtained by means of Python scripts that continuously polled Twitter’s streaming application programming interface. This service provides a sample stream of data based on key terms and hashtag searches. Tweets were collected between December 24, 2016, and April 21, 2017. The key terms used to collect the tweets included e-cigarette, vaping, etc (see Multimedia Appendix 1 for complete list). The key terms could have appeared in the post or in an accompanying hashtag (ie, vaping or #vaping). The university’s institutional review board approved all procedures.

The terms used to collect tweets during the study period resulted in an initial corpus of 6,185,153 tweets. However, Twitter has quickly become subject to third-party manipulation where computer algorithms designed to automatically produce content and engage with legitimate human accounts on Twitter (social bots) are created to influence discussions and promote specific ideas or products [25]. Social bots are meant to appear as genuine human users operating Twitter accounts; their profiles are often complete with metadata (name, location, pithy quote) and a photo/image. Social bots on average generate more tweets than the average human user. Therefore, social bots are producing more content on a topic. Social bots make indiscriminate references to an array of content while at the same time perpetuating select conversations, giving the appearance that a specific topic is more prominent than it is offline. Their adoption has been documented in a variety of domains, including political astroturfing [26], stock market manipulation [27], spread of misinformation [28], promotional content [29], and in sentiment classification [24].

In order to distinguish between human users and social bots, certain criteria such as information diffusion patterns (based on retweets or mentions), friend features (for example, ratio of followers to followees), content (frequency of nouns/verbs/adverbs in a tweet), and sentiment features (emotion scores) are used. The BotOrNot algorithm combines these features to obtain a single score between 0 and 1 that indicates if a Twitter account is a social bot or not [28,30]. Evaluations of the BotOrNot program have shown that an account is most likely to be a bot if the account score is ≥0.6 [24,27,29]. The method used for bot detection has a detection accuracy above 95%, suggesting that error from inappropriate removal of legitimate accounts is minimal [25]. Spam-specific, unrelated to e-cigarettes, tweets were manually removed based on occurrence of certain keywords (see Multimedia Appendix 1 for complete list). Among the 6,185,153 tweets, 3,994,481 (64.58%) were identified as spam and were removed leaving 2,190,672 tweets remaining in the clean dataset. About a quarter of these tweets, 412,816, contained at least 1 hashtag, yielding 119,964 unique hashtags. Hashtags provide useful information to identify topics of conversation.

To identify topics of e-cigarette–related conversations, we created a Twitter hashtag co-occurrence network and identified co-occurring clusters of hashtags. The concept clusters are built as follows: the network nodes represent all the different hashtags extracted from the tweets, and for each tweet that contains more than 1 hashtag, an edge (link) is placed between the nodes corresponding to the co-occurring hashtags. A weight is associated to each edge to convey the number of co-occurrences. The weighted network that emerges from this procedure is then plotted using the network visualization tool Gephi [31] and inspected to learn which topics are often discussed together.

Given the volume of data, we used specific network conditions to filter the visualized clusters. Among Gephi’s visualization algorithms, we choose the Fruchterman Reingold force-directed layout [32]. The algorithm works in analogy to gravity forces in natural systems: 2 nodes attract each other based on the strength of their interaction (ie, the weight of their link). This type of layout maximizes readability of network visualizations by minimizing node overlap. Given the scale of the Twitter hashtag co-occurrence network, to limit the number of nodes to display, we imposed a filter to hide nodes with low degrees. This filtering process allows us to focus on the most important clusters and nodes, namely those that co-occur more frequently. From Gephi’s algorithms we finally used the Louvain community detection algorithm which is used to reveal the most significant clusters, groups of nodes tightly interconnected [33]. In order to illustrate how results can change due to social bots, we created and inspected the concept clusters from the 2 corpora of tweets, respectively including and excluding social bots and their tweets. We then computed odds ratios from 2×2 contingency tables ([occurrence of specific hashtag among social bots/occurrence of specific hashtag among humans]/[occurrence of all other hashtags among bots except the specific hashtag/occurrence of all other hashtags among humans except the specific hashtag]) to detect if hashtags varied by source using a Fisher exact test to determine statistical significance.

To identify where in the United States e-cigarette–related discussions were taking place, we extracted the user location from the geographic coordinates field of each tweet, which Twitter collects automatically. However, we observed that many tweets did not have the coordinates defined because each individual Twitter account can elect to turn off this function on their mobile phone, device, or computer, preventing Twitter from collecting this information. To overcome this limitation, we translated the location entered by the user in their metadata (eg, Los Angeles) to latitude and longitude coordinates. Given these 2 strategies, we could identify user location for approximately 1% of all users in the analytical sample, representing 36,549 users in the United States. We used a heat map plot to determine where individuals discuss e-cigarettes. In a heat map, stronger color intensity (similarly to heat) suggests higher intensity of use in a specific area and vice versa. By looking at frequency of tweets by location we can see where priority areas exist for e-cigarette education campaigns.

## Results

The cluster analysis from the corpus of hashtags from human users contained 238 specific hashtags or nodes and 5203 edges (Multimedia Appendix 2). Cluster 1 (pink) contained hashtags indicative of behaviors (eg, #vaping), vaping identity (eg, #vapelife), and vaping community (eg, #vapenation) (Table 1). Cluster 2 (green) contained hashtags indicative of vaping products (eg, #eliquids), vaping identity, and vaping community. Cluster 3 (orange) contained hashtags indicative of dual tobacco use (eg, #hookah) and polysubstance use (eg, #marijuana).

**Table 1:** Most common hashtags in each respective cluster from the bot-free corpus.

Cluster^a^	Hashtags
1 (pink)	vaping, ecigs, vapelife, vapeporn, weed, buzz, vaporizer, vapenation, eliquid, cannabis, vape, vapes, bigtobacco, ejuice, smokeshop
2 (green)	eliquids, vaper, vapelife, smoke, instavape, vapecommunity, ecig, vapors, atomizer, vapeclub, vapestagram, vapesociety
3 (orange)	smokers, nowsmoking, cigaretters, tobacco, week, marijuana, cigars, whisky, scotch, smoker, cigarettes, hookah, addiction, blu

^a^Colors correspond to the figure found in Multimedia Appendix 2.

**Table 2:** Most common hashtags in each respective cluster from the bot corpus.

Cluster^a^	Hashtags
1 (orange)	Cigars, cigar, blu, tobacco, cigarette, smoke, smoking, photography, galanecigars, lifelove
2 (gray)	vapes, vape, vaping, vapor, ecig, ecigs, vapefam, vapelife, vapor, smok, vaporstorm, eliquids, vapepen, vapefamily, vapeshop, vapecommunity, vapeporn, vapers, vaporizer, ecigaretters
3 (blue)	esmoke, esmoking, online, beast, mod, cheap, cigpet, starterskit, esmoker, mobile, ismog, modbox
4 (green)	marijuana, smoking, health, weed, tobacco, cannabis, cbd, thc, cool, bongs, tobacco, quality, cheap, vapes, ejuice, quitsmoking

^a^Colors correspond to the figure found in Multimedia Appendix 3.

**Table 3:** Associations between hashtags and data source (bots vs humans coded) with an odds ratio > 1 indicating greater likelihood from a bot.

Hashtags	Odds ratio	*P* value
addiction	0.62	.006
atomizer	0.53	<.001
beast	2.72	<.001
bigtobacco	0.08	<.001
blu	1.33	<.001
bongs	1.79	<.001
tobacco	1.05	.002
buzz	0.66	<.001
cannabis	1.81	<.001
cheap	1.81	<.001
cigar	0.66	<.001
cigarette	1.65	<.001
cigarettes	0.55	<.001
cigars	0.54	<.001
cigpet	2.73	<.001
cool	1.58	.03
ecig	0.54	<.001
ecigs	1.52	<.001
ejuice	0.97	.23
eliquid	0.73	<.001
eliquids	1.06	.40
esmoke	2.88	<.001
esmoking	2.87	<.001
esmoker	2.89	<.001
health	1.00	.92
hookah	0.29	<.001
instavape	0.80	.03
ismog	2.89	<.001
marijuana	1.25	<.001
mobile	1.68	<.001
mod	2.47	<.001
modbox	2.41	<.001
nowsmoking	0.70	.003
online	2.78	<.001
photography	0.96	.87
quality	1.80	<.001
quitsmoking	2.27	<.001
scotch	0.02	<.001
smoke	1.00	.85
smoker	2.61	<.001
smokers	1.66	<.001
smokeshop	1.33	.07
smoking	1.04	.31
starterskit	2.88	<.001
thc	2.43	<.001
tobacco	1.05	.002
vape	0.82	<.001
vapecommunity	0.19	<.001
vapefam	0.34	<.001
vapefamily	1.42	<.001
vapelife	0.63	<.001
vapenation	0.78	<.001
vapepen	0.89	.03
vapeporn	0.90	<.001
vaper	1.86	<.001
vapers	1.67	<.001
vapeshop	1.13	.03
vapesociety	0.34	<.001
vapestagram	0.69	<.001
vaping	1.15	<.001
vapor	0.68	<.001
vaporizer	0.29	<.001
vapors	0.52	.0002
vaporstorm	2.94	<.001
weed	1.03	.5694
whiskey	0.02	<.001

The cluster analysis from the corpus of hashtags from social bots contained 4 clusters with 137 hashtags or nodes and 1600 edges (Multimedia Appendix 3). Cluster 1 (orange) contained hashtags indicative of behaviors and dual tobacco use (Table 2). Cluster 2 (gray) contained hashtags indicative of behaviors and vaping identity and vaping community. Cluster 3 (blue) contained hashtags indicative of products (eg, #starterskit, #modbox), including brand new products (eg, #ismog, a new smart device with touch technology on a vaping box, #cigpet, a new high wattage tank or “super tank”). Cluster 4 (green) contained hashtags indicative of smoking cessation (eg, #quitsmoking), interest in health (eg, #health), and polysubstance use.

Social bots were more likely to post hashtags that referenced smoking cessation and new e-cigarette devices compared to human users (Table 3). For example, social bots were significantly more likely to post #quitsmoking, #ismog, and #cigpet compared to human users.

**Figure 1:**
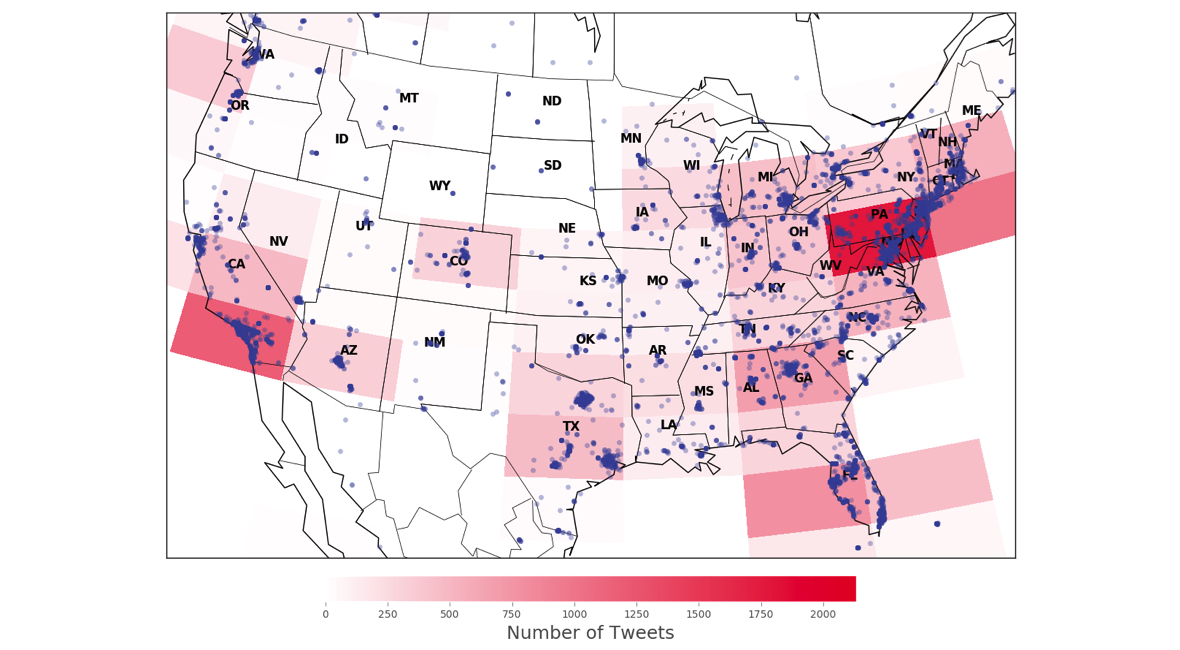
Heat map from December 25, 2016, to April 21,2017, for 26,565 tweets.

The heat map representing 26,565 tweets collected from December 24, 2016, to April 21, 2017, shows that the volume of tweets is highest in the Mid-Atlantic (eg, Pennsylvania, New Jersey, Maryland, and New York) and high on the West Coast and Southwest (eg, California, Arizona and Nevada) (Figure 1).

## Discussion

### Principal Findings

Data from online social systems may be used to complement and extend the surveillance of health behaviors including tobacco product use. The hashtags we studied here provide several direct insights into e-cigarette–related attitudes and behaviors with the identification of 3 clusters that represent the most cohesive posts. The cluster analysis from the corpus of hashtags from human users demonstrated the existence of a vaping identity and vaping community. Use of these hashtags may serve further internalization of, and social bonding around, vaping-related identities. These hashtags also suggest discussions of vaping may occur in an echo chamber on Twitter in which ideas and beliefs are amplified by those in the network [34], normalizing vaping.

In the cluster analysis from the corpus of hashtags from human users, we found many references to vaping-related products. These hashtags represent a way for commercial users to make their posts searchable and integrate themselves into online communities of vapers. Noncommercial users may also use these hashtags to communicate to their followers which products they recently purchased or which products they like to use together (eg, their favorite modifiable device paired with their favorite e-liquid) [10].

The third hashtag cluster found in the corpus from human users indicated dual tobacco use and polysubstance use. These co-occurring hashtags may reflect a syndrome of risky behavior among select vapers. While research is accumulating about dual e-cigarette and cigarette use [35,36], there is a dearth of research on the associations between vaping and hookah, marijuana, alcohol, and other substance use. The findings from this study should spur efforts to investigate these associations further. When the population-level impact of e-cigarettes is being debated, the co-occurrence of vaping with alcohol and other substances should also be considered.

In the corpus of hashtags from social bots, several results stood out in contrast to the results from the human user corpus. For one, a cluster of hashtags was detected that referenced smoking cessation. This suggests social bots may be used to perpetuate discussions on e-cigarettes as a cessation device. While earlier research has suggested Twitter posts about vaping referenced the use of e-cigarettes in cessation [22], it is important to distinguish between individual users and social bots when analyzing posts on Twitter [23,24,37]. Social bots may perpetuate misinformation about the efficacy of e-cigarettes in cessation, thus requiring education campaigns to serve as a vehicle to correct this misinformation.

Hashtags from social bots also represented newly introduced products to the marketplace (eg, #ismog and #cigpet) which were significantly less prevalent in the human user corpus of hashtags. This finding highlights a clear benefit of using social media data in public health surveillance. In addition to searching for known keywords and observing trends in the number of social media posts that contain those keywords, the concept cluster analysis can identify new keywords or hashtags posted on Twitter. This process can serve as an early warning system informing public health researchers about new products or new ways in which products are appealing to the public. By using social media data and keyword co-occurrence analyses we can identify new products (like ismog or cigpet), brands, marketing themes, activities, and events associated with tobacco product use as they emerge in near real time. The findings from this study complement recent research that relied on search navigation data to detect growing interest in heat-not-burn tobacco products [38]. Taken together, public health researchers could use data from online social systems to fill knowledge gaps quickly and respond more readily to the populations they serve.

Most posts were from the Mid-Atlantic and Southwest, which is compatible with earlier research relying on search navigation data [9]. The findings mark priority areas for e-cigarette education campaigns. Social media may be one way to engage with nonusers of tobacco products to inform them of the addictive properties of nicotine as well as the harms of e-cigarette use [39]. Using social media as a complementary surveillance system could allow public health researchers to identify geographic disparities in emerging tobacco product use earlier than traditional methods. While Twitter data should not be used to supersede traditional health behavior surveillance systems, social media could be used to fill information gaps quickly and can provide an important starting off point to address an issue of great import to public health or policy.

### Limitations

Data collection relied on Twitter’s streaming application programming interface, which prevents collecting tweets from private Twitter accounts. As a result, findings may not represent the attitudes and behaviors from individuals with private accounts. This study used hashtags to identify themes in posts on Twitter but did not specifically read and interpret each post that the hashtags accompanied. Additional valuable information could have been learned from the content of the posts that was not described herein. Approximately 1% of all users in the analytical sample provided data that allowed us to describe the geographic areas in which e-cigarette–related discussions took place in the United States. While this is a small percentage, it is compatible with earlier work [25,40] and represents 36,549 users in the United States. Additionally, we did not have the necessary demographic information (eg, age) of Twitter users to consider population density and age distributions of geographic areas.

### Conclusion

The findings from this study can inform the design of public health surveillance in the future. This study demonstrated the utility in using social media data in understanding attitudes and behaviors and the importance of distinguishing between Twitter posts from social bots and humans during this process if the intent is to assess views held by real users. Findings should spur efforts to better understand the consequences of e-cigarette–related discussions on Twitter.

## Appendix

Multimedia Appendix 1 Key search terms.

Multimedia Appendix 2 Concept cluster built from hashtags collected from a bot-free corpus.

Multimedia Appendix 3 Concept cluster built from hashtags collected from a bot corpus.
