# Supplementary material for: E-Cigarette Surveillance With Social Media Data: Social Bots, Emerging Topics, and Trends
Source: JMIR Public Health Surveill. 2017 Dec 20;3(4):e98. doi: 10.2196/publichealth.8641 (PMC5752967; doi:10.2196/publichealth.8641)
Supplement: Multimedia Appendix 1 [file publichealth_v3i4e98_app1.pdf]

List of keywords

Twitter_Keywords=['e-cig', 'ecig','e-cigs','ecigs','e-cigarette','ecigarette','e-cigarettes','ecigarettes','vape','vaper','vaping','vapes','vapers','nicotine','tobacco','cigarette','cigarettes','cigar','atomizer','atomizers','cartomizer','cartomizers','ehookah','e-hookah','ejuice','ejuices','e-juice','e-juices', 'eliquid', 'eliquids','e-liquid','e-liquids','blu','njoy','green smoke','south beach smoke', 'eversmoke', 'joye510','joye510','joyetech','lavatube','lavatubes','logicecig','logicecigs','smartsmoker','smokestiks','v2 cig','v2 cigs','v2cigs','v2cig','mistic','21st century smoke','logic black label','finiti','nicotek','cigirex','logic platinum','cigalectric','xhale o2','cig2o','green smart living','krave','secondhand vape','secondhand vaping','second-hand vape','second-hand vaping','vape smoke','ecig smoke','e-cig smoke','e-cigarette smoke','vape shs','ecig shs','vape secondhand smoke','vape second-hand smoke','esmoke','e-smoke','stillblowingsmoke','still blowing smoke','notblowingsmoke','not blowing smoke','capublichealth','tobaccofreekids','notareplacement','trulyfree','truly free','sb140','sb 140','sb24','sb 24','cherry tip cigarillos','mini-cigarillos','tip cigarillos','king edward cigars','royal gold cigars','sweet coronella','swisher blk','swisher sweets','vapercon','vapercon west','grimmgreen','vapor','electronic cigarette','vape meet','EcigsSaveLive','EcigsSaveLives','EcigsSavesLives']

List of Spam words

blacklist = set(['deal', 'shop', 'promo','win', 'dvd', 'movies', 'giveaway', 'deals', 'horror', 'bluray', 'ebay', 'gameofthrones', 'movie','deal', 'auction', 'charlestonshooting', 'fiftyshades', 'entertainment', 'news', 'ultimatepizzahit','shoes', 'nowplaying', 'sale', 'music', 'competition', 'tv', 'actor', 'fashion', 'actress','hungergames', 'twilightsaga', 'onsale', 'rt', 'celebs', 'sandrabland', 'bestbuy', 'wwe', 'twilight','forsale', 'katypwtl', 'thewalkingdead', 'actionmovies', 'soundcloud', 'amazon', 'johonnydepp', 'nike','ageofultron', 'insurgent', 'porn', 'shopping', 'buynow', 'reddit', 'android', 'disney', 'bargains','discount', 'thehobbit', 'film', 'gossip', 'leonardo', 'romanticmovies', 'jenniferlawrence', 'furious7','dyinglight', 'action', 'ad', 'bighero6', 'sex', 'bestseller', 'chappiemovie', 'ericgarner', 'pitchperfect2','code', 'promo', 'free', 'shipping', 'order', 'dvdbluray', 'vicecityatl'])
