# Supplementary figures and images for: E-Cigarette Surveillance With Social Media Data: Social Bots, Emerging Topics, and Trends
Source: JMIR Public Health Surveill. 2017 Dec 20;3(4):e98. doi: 10.2196/publichealth.8641 (PMC5752967; doi:10.2196/publichealth.8641)

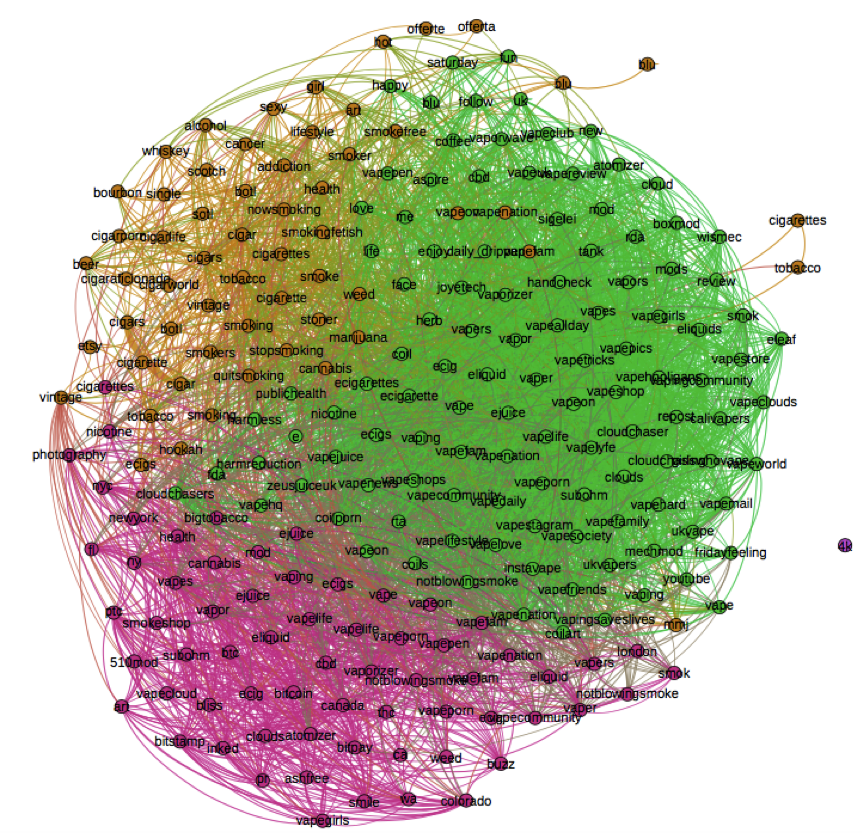

Supplement: Multimedia Appendix 2 [file publichealth_v3i4e98_app2.png]

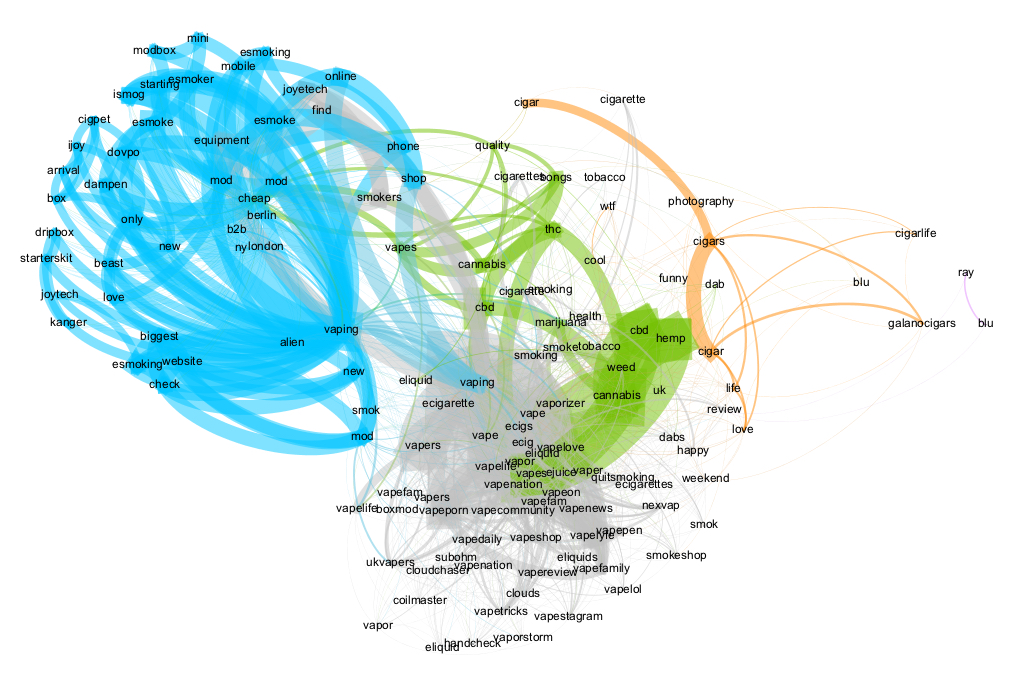

Supplement: Multimedia Appendix 3 [file publichealth_v3i4e98_app3.jpg]
